# Supply-Side Constraints Are Insufficient to Explain the Ontogenetic Scaling of Metabolic Rate in the Tobacco Hornworm, *Manduca sexta*

**DOI:** 10.1371/journal.pone.0045455

**Published:** 2012-09-19

**Authors:** Viviane Callier, H. Frederik Nijhout

**Affiliations:** Biology Department, Duke University, Durham, North Carolina, United States of America; University of Sydney, Australia

## Abstract

Explanations for the hypoallometric scaling of metabolic rate through ontogeny generally fall into two categories: supply-side constraints on delivery of oxygen, or decreased mass-specific intrinsic demand for oxygen. In many animals, supply and demand increase together as the body grows, thus making it impossible to tease apart the relative contributions of changing supply and demand to the observed scaling of metabolic rate. In larval insects, the large components of the tracheal system are set in size at each molt, but then remain constant in size until the next molt. Larvae of *Manduca sexta* increase up to ten-fold in mass between molts, leading to increased oxygen need without a concomitant increase in supply. At the molt, the tracheal system is shed and replaced with a new, larger one. Due to this discontinuous growth of the tracheal system, insect larvae present an ideal system in which to examine the relative contributions of supply and demand of oxygen to the ontogenetic scaling of metabolic rate. We observed that the metabolic rate at the beginning of successive instars scales hypoallometrically. This decrease in specific intrinsic demand could be due to a decrease in the proportion of highly metabolically active tissues (the midgut) or to a decrease in mitochondrial activity in individual cells. We found that decreased intrinsic demand, mediated by a decrease in the proportion of highly metabolically active tissues in the fifth instar, along with a decrease in the specific mitochondrial activity, contribute to the hypoallometric scaling of metabolic rate.

## Introduction

The scaling of metabolic rate with body size has been the subject of many empirical and theoretical studies [1], [2], [3], [4], [5], [6], [7], [8], [9], [10], [11], [12], [13]. The scaling of metabolic rate with body mass commonly resembles a power-law relationship that can be described by the equation: metabolic rate = a*mass^b^. If metabolic demand were strictly proportional to body mass, then the exponent, b, would be 1; in contrast, if metabolic rate were a function of the rate at which energy is lost from surface area, then b would be 2/3. Kleiber [14], [15] found that the scaling exponent for several species over a large range of sizes was ¾, which does not neatly match either hypothesis. Although there is substantial controversy over the exact value of the scaling exponent [8], [10], [16], [17] and consequently which model presents the most accurate fit to the data, two certainties remain: (1) hypoallometric scaling of metabolic rate with body mass (0<b<1) is far more common than isometric (b = 1) or hyperallometric (b>1) mass scaling, and (2) the mechanisms responsible for metabolic allometries are incompletely understood.

West, Brown and Enquist (1997) (WBE) argued that the hypoallometric scaling of metabolic rate is a necessary consequence of fractally branching supply networks. They derive the ¾ scaling exponent based on the primary (albeit somewhat implicit) hypothesis that the transport of rate-limiting metabolites constrains nutrient usage due to the geometry of a space-filling distribution network with the following properties: the terminal branches of the fractal supply network are invariant with body size, the energy required to circulate fluid through the system is minimized, and the volume of the network occupies a constant proportion of the total body volume. Variations on this modeling approach that preserve the same fundamental supply-limiting hypothesis have been proposed [6], [7], [18], showing that the ¾ law could emerge from other (non-fractal) network architectures. The supply-based constraint hypothesis assumes that the metabolic rate is somehow restricted by the rate at which the material needs for metabolism can be supplied and that this constraint results in the observed hypoallometric relation between mass and metabolic rate.

Others have argued that metabolic rate is constrained to scale hypoallometrically due to the geometry of cell size [8], [9]. Since a large fraction of cellular ATP is used is to maintain ion gradients across membranes, this model hypothesizes that metabolic allometries may be caused by scaling of net cell membrane surface area with body volume. When body size increases through an increase in cell size, the cell surface-to-volume ratio decreases; therefore a unit of body mass will contain relatively less cell membrane area and the mass-specific metabolic rate should decrease. If size expansion is realized exclusively through cell size, then the standard metabolic rate should increase in proportion to body volume to the 2/3 power. If body size increased solely through an increase in cell number, then the metabolic rate per unit of body mass should stay identical, and total metabolic rate should scale proportionally with body mass. This model predicts a range of scaling exponents since growth is frequently due to a combination of increase in cell size and increase in cell number, so metabolic rate is expected to scale with an exponent between 2/3 and 1.

**Figure 1:**
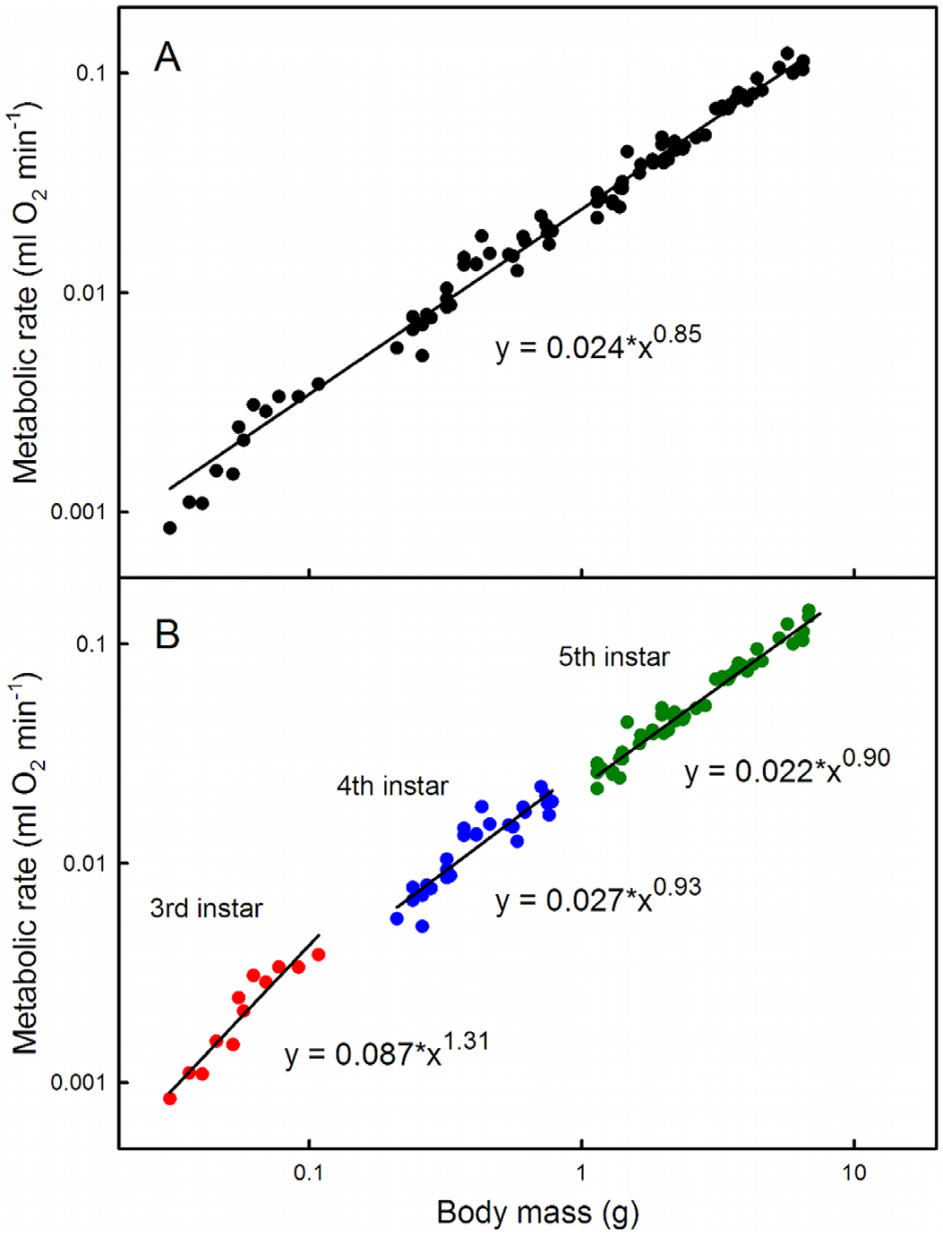
Metabolic scaling across and within larval instars of *Manduca*. Data are plotted for oxygen consumption rates prior to the critical weight in each instar. A) Measured across instars 3 to 5, metabolic rate scales hypoallometrically as mass 0.85. B) Metabolic rates scale differently in individual instars, with an apparent decrease in scaling in successive instars.

These authors suggested that metabolic rate is driven by cell-membrane-dependent processes, and that the scaling of metabolic rate is determined by the changes in the proportion of membranes per unit volume. Chown et al. (2007) found that scaling exponents varied from 0.67 to 1.0 in their survey of 391 insect species from 16 Orders, using the size of the eye as a proxy for body size, and using cells of the eye to assess the contributions of cell size and cell number to size. They noted that in species in which body size variation was due to variation in cell number, the metabolic rate scaled isometrically with body mass, whereas in species where both cell size and cell number contributed to variation in body size, the scaling exponents for metabolic rate were less than 1. Thus, the Kozlowski hypothesis is consistent with intra-specific patterns of metabolic scaling considering a very specific tissue, the insect eye. Nevertheless, the Kozlowski model does not take into account that different cell types may have different metabolic demands that are independent of cell size. The hypothesis also depends on the assumption that the vast majority of metabolic demand is driven by processes at cell surfaces, as opposed to internal processes such as energy metabolism, maintenance, the production and turnover of proteins or replication of DNA. In addition, this hypothesis does not consider that cell membranes are seldom smooth envelopes.

Other hypotheses about demand-driven metabolic rates have been proposed [19], [20]. Ricklefs [20] proposes that growth rate (and by extension, metabolic rate) reflects the growth potential of tissues for cell proliferation and growth, which decrease as the tissues achieve functional maturity; metabolic rate is not limited by resource supply, but by tissue growth potential. Although this argument is plausible, there is no straightforward way to measure “tissue growth potential”. Another hypothesis for demand-driven metabolic rate was proposed by Speakman and Krol [19], who argue that metabolic rates are constrained by maximal capacity to dissipate body heat. Essentially, this is the flip-side of the argument that smaller animals must have a higher specific-metabolic rate because they have a higher surface-area-to-volume ratio, and therefore lose heat faster: Speakman and Krol (2011) instead argue that large animals can’t lose heat fast enough. Although this argument may apply to endotherms, it probably does not apply to growing insect larvae and other ectotherms.

There is controversy over whether supply-side constraints [6], [12] or intrinsic metabolic demand [9], [20] determine the observed scaling relationship of metabolic rate with body mass. In most animals, supply capacity and demand increase simultaneously as the body grows [21], [22], so it is difficult to tease apart the contributions of each to the observed metabolic rate.

In insect larvae, the body (which consumes oxygen) grows continuously but the tracheal system (which supplies oxygen to each cell) grows discontinuously: it is fixed within a larval instar and increases in size only at each molt [23]. The volume of the tracheal system might even decrease during an instar due to compression by the growing metabolically active tissue [23], [24]. Reduced conductivity is reflected by an increased critical pO_2_ within an instar [24], [25]. Thus the growth of oxygen supply structures (the tracheal system) is decoupled from that of the tissues that demand oxygen. Because of this mode of growth, insects are particularly well suited to study the effects of growth-induced and size-dependent changes in supply and demand on metabolic rate.

**Figure 2:**
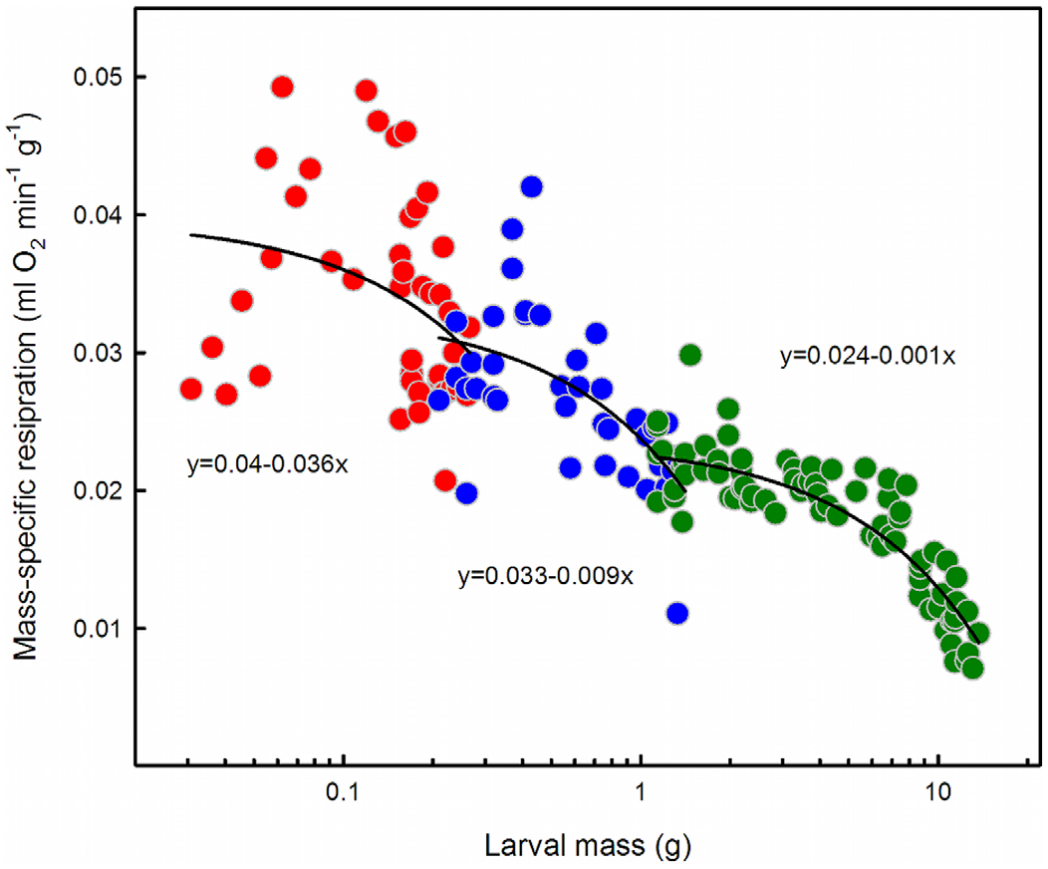
The mass-specific metabolic rate decreases within and between instars (although the within instar decrease is not significant in the third instar). At the beginning of each instar, the supply structures are not constraining, so this decrease is most likely due to a decrease in intrinsic oxygen demand. In the second half of the fifth instar there is a strong drop-off in the size-specific metabolic rate, which suggests that post-critical weight larvae may be oxygen-limited.

In *Manduca sexta* larvae, respiration rate increases in tight correlation with increasing body mass early in each instar, but levels off and becomes constant after a larva passes the critical weight even though larval mass continues to increase [23]. This observation suggests that larvae become oxygen limited in the latter part during each instar [23], [24]. Early in each instar, supply capacity (represented by the dimensions of the tracheal system) exceeds demand (represented by body mass). Thus at the beginning of each instar the metabolic rate is primarily driven by the intrinsic demand for oxygen, which explains the linear relationship between mass and respiration rate before the critical weights (Figure 1). As the larva grows within an instar, demand for oxygen increases, without a concomitant increase in supply capacity. Larval metabolism thus becomes increasingly constrained, and the leveling off of the metabolic rate after the critical weight represents the limit of the constraint on metabolic rate, as shown in [23]. When a larva next molts, the tracheal system enlarges by a discrete amount, and the metabolic rate once again represents the intrinsic demand of the tissue, unconstrained by supply.

Sears et al. [13] have conducted a thorough analysis of the ontogenetic scaling of growth, metabolism and assimilation in *Manduca* to determine whether assimilation of nutrients becomes limiting for growth due to changes in the geometry of gut surface area as the animal grows. They showed that metabolic scaling changes during larval development and that the metabolic rate changes with the same exponent as the growth rate. In the present paper, we focus on determining the relative contributions of oxygen supply and demand to the observed metabolic scaling pattern. First we show how metabolic rate and mass-specific metabolic rate scale with body size as larvae grow through several larval instars. We show that the mass-specific metabolic rate declines from instar to instar, and we examine the possible roles of differential tissues metabolism and declining mitochondrial activity that could explain this effect.

## Methods

### Respiration Measurements

Respiration rates in third, fourth, and fifth instar larvae were measured as described in [23]. Oxygen consumption rates were measured using a constant-pressure respirometer in a temperature-controlled room at 25°C. The respirometer was constructed from a 15 ml test tube with a 1 ml pipette inserted in a one-hole stopper. A drop of colored water served as the volumetric marker. Carbon dioxide was absorbed by a wad of paper towel soaked in a 20% solution of KOH. A control tube, identical in all respects except for the presence of an experimental animal, served as a monitor for effects of variation in atmospheric pressure and vessel temperature. After inserting a larva in an experimental tube and sealing the control tube we waited until the volume of the control tube had stabilized, and then measured the rate of oxygen consumption in the experimental tubes at 1 minute intervals to the closest 0.01 mL. The respiration rate was measured for 1–2 hours for the third instars, 0.5–1 hour for the fourth instars, and 15–20 minutes for the fifth instars. In all cases there was no change in the volume of the control tubes during the measurement period.

**Figure 3:**
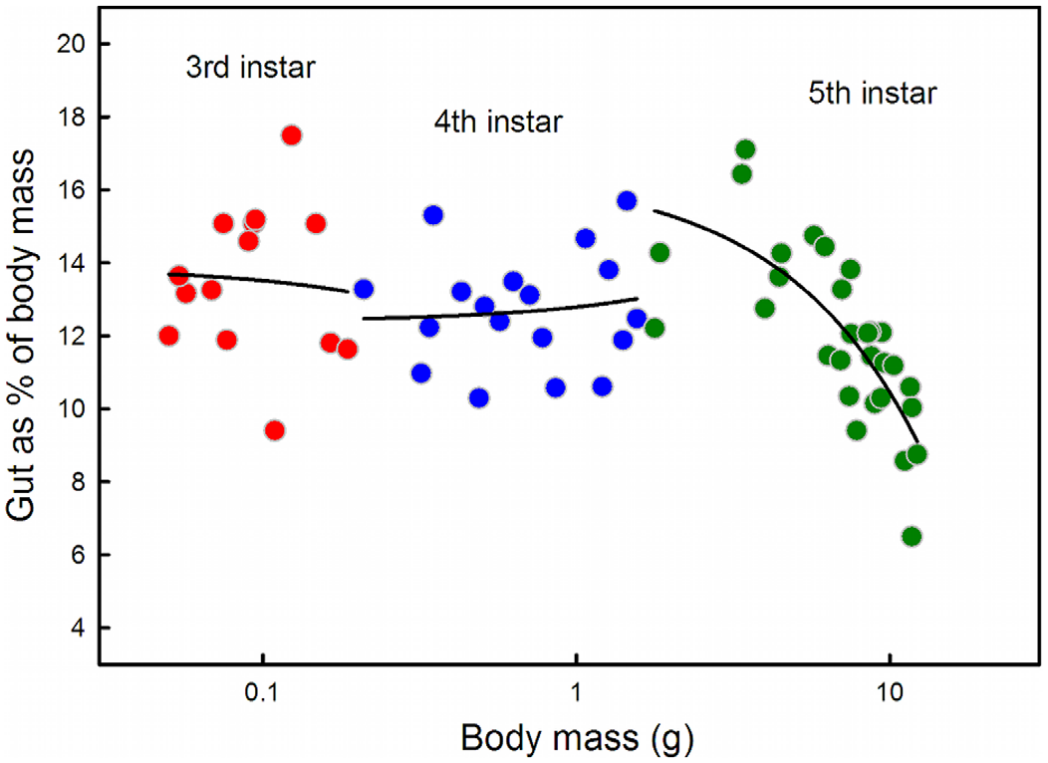
Midgut as a proportion of total body mass is constant during the 3rd and 4th larval instars and declines during the 5th instar.

### Tissue Measurements

To determine the proportion of gut mass relative to total body mass, we determined the dry weight of the (empty) gut and carcass (body without gut) of larvae from the third, fourth and fifth instars. Larvae of each instar were anesthetized under CO_2_, weighed and dissected. The gut was removed, emptied of its contents by rinsing in water, dried with paper towels, and weighed to the nearest 0.01 g. The wet weight of the carcass (without the gut) was also recorded. The gut and carcass were then placed on a tared aluminium foil and placed in a drying oven at 65°C until the weight stabilized (typically about 48 hours). The dry weights of guts and carcasses were recorded to the nearest 0.1 mg.

### Mitochondrial Isolation and Cytochrome Oxidase (COX) Assay

To determine the contribution of mitochondrial activity to the decrease in mass-specific metabolic rate of *Manduca*, we used a mitochondrial isolation and COX activity assay (BioChain kits # KC010100 and #KC310100) and followed the instructions in the kit (the technique is also described by [26]). In brief, larvae from the beginning of the third, fourth and fifth instars were weighed and then anesthetized with CO_2_ and dissected. The gut was removed, emptied and rinsed, and dried by wicking off liquid with a paper towel. A sample of 70 mg wet weight was rinsed in PBS for 10 minutes, minced to smaller pieces with a razor blade, and homogenized with a glass homogenizer. 1 mL of mitochondria isolation buffer and homogenate were added to a 1.5 mL Eppendorf tube. Tissue was disrupted by sonication for 30 seconds using a microtip on a Fisher 550 Sonic Dismembranator. The samples were centrifuged at 1000 rpm in a MicroSpin AccuR refrigerated centrifuge (Fisher) for 12 minutes. The pellet was discarded, and the supernatant was then centrifuged at 13000 rpm for 16 minutes. The pellet was collected and resuspended in 500 µL mitochondria isolation buffer, and the previous centrifugation steps were repeated. The final pellet was resuspended in 20 µL mitochondrial storage buffer. 10 µL of each sample was set aside for the protein assay, and 100 µL lysis buffer with 1× protease inhibitors was added to the remaining 10 µL. This technique is not guaranteed to isolate all mitochondria in a sample, nor is it certain there is no cellular debris in the pellet, thus the protein content is not an accurate measure of the quantity of mitochondria but rather a measure of the sample size.

COX activity was calculated according to the BioChain protocol by measuring the initial rate of change in absorbance (using a Shimadzu UV-2401PC spectrophotometer), and accounting for dilution and sample volume. Mass-specific COX activity was calculated by dividing COX activity by body mass, as done by [26]. Protein-specific COX activity was calculated by dividing COX activity by the measured protein content of the sample.

### Protein Assay

Protein was determined by the BCA Protein Assay Kit (Pierce Product #23223; Rockford, IL), using a dilution series of bovine serum albumin (BSA) as the standard. 200 mL of the BCA working solution were added to each tube with 10 mL sample and incubated at 37 degrees C for 30 minutes. Absorption was read at 562 nm and protein content was calculated by reference to a standard (BSA) curve.

**Figure 4:**
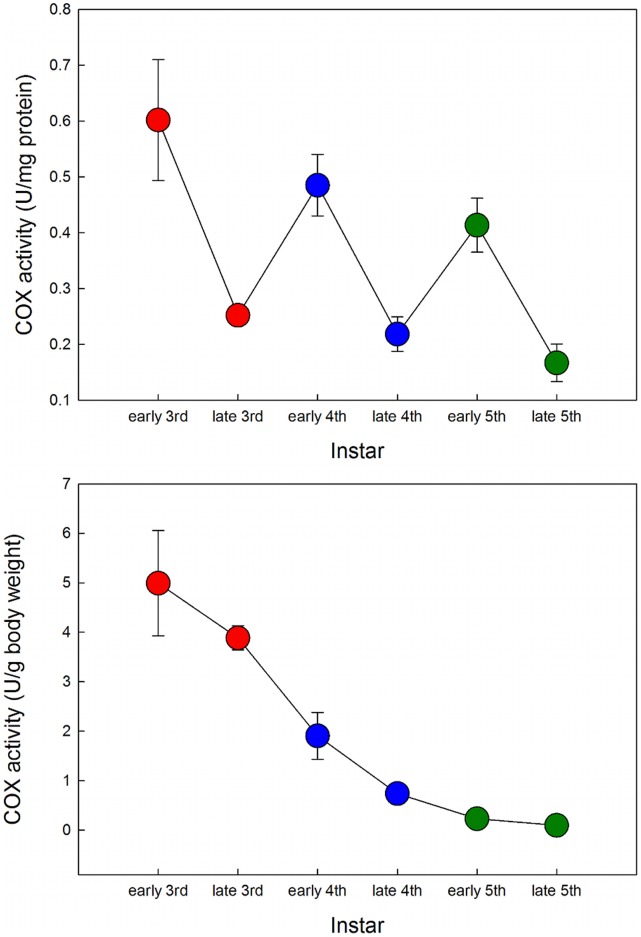
The COX activity per unit of protein decreases within an instar, but increases again at the next instar. However, COX activity per unit mass decreases monotonically across instars, suggesting that intrinsic demand decreases as the larva grows.

## Results

### Ontogenetic Scaling of Metabolic Rate

The respiration rates of *Manduca* larvae in three successive instars are shown in Fig. 1. An early third instar larva weighs approximately 0.03 grams and consumes approximately 0.001 mL O_2_ per minute; an early fifth instar weighs on the order of 1.2 grams and consumes on the order of 0.02 mL O_2_ per minute. Although the respiration rate for the combined third, fourth and fifth instars appears to scale as mass 0.85 (Fig. 1A), within each instar the metabolic rate before critical weight scales as mass 1.31 for the 3rd instar, mass 0.93 for the 4th instar, and mass 0.91 for the 5th instar (Fig. 1B). The pre-critical weight, within-instar scaling exponents are higher than the whole-instar scaling exponents measured by [13]; the difference is due to the fact that metabolic rate levels off after critical weight in each instar [23], so the exponent is lower when the post-critical weight data are included. Our data are consistent with their finding that the ontogenetic scaling model parameters change from instar to instar.

If the intrinsic metabolic demand of cells and tissues remained constant, then one would expect the mass-specific metabolic rate to reset to the same value early in each instar, because at that time there is no constraint on supply: oxygen supply structures appear to be overbuilt for the metabolic demands early in each instar, as evidenced by low critical pO_2_ values in early-instar larvae relative to higher critical pO_2_ values in late-instar larvae [24], [27]. We would expect that pre-critical weight larvae are not oxygen-constrained and therefore that their mass-specific metabolic rate should reflect intrinsic oxygen demand. Although there was no *a priori* reason to expect instrinsic oxygen demand to change, we found that the mass-specific metabolic rate prior to the critical weight also decreases from instar to instar (Fig. 2).

This finding suggests that supply-side constraints alone cannot explain the hypoallometric scaling of metabolic rate. Instead that there is a progressive decrease in the mass-specific intrinsic demand for oxygen. There are two (not mutually exclusive) ways in which the mass-specific metabolic demand could decrease as the larva increases in size: the proportion of highly metabolically active tissues relative to total body size could decrease, and/or the intrinsic metabolic demand of all cells could decrease.

In *Manduca* larvae the midgut is the most metabolically active tissue [28], [29], and constitutes a significant proportion of the total metabolic tissue of the larva (Figure 3). The midgut is also a large and accessible organ whose mass can be accurately measured and therefore presents us with a good system in which to study the relative contribution of a specific tissue to the changing overall metabolic demand.

The activity of mitochondria can be estimated by measuring the activity of cytochrome c oxidase (COX), a mitochondrial enzyme that is a reliable indicator of a tissue’s metabolic potential [30] and is required for normal growth and development in insects [31]. We measured COX activity in the midgut during the third, fourth and fifth larval instars. In figure 4 we plot specific COX activity in two ways. Figure 4A shows activity per µg protein, and Figure 4B shows activity per gram of body weight, as done in [26]. By both measures COX activity decreases throughout ontogeny but they show different patterns. Protein-specific COX activity drops during the instar and then goes up again at the beginning of the next instar, but not to the same relative level as at the beginning of the previous instar (Figure 4A). By contrast, body mass-specific COX activity gradually declines within an instar but also from instar to instar, though at a decreasing rate (Figure 4B). This finding suggests that the decrease in size-specific metabolic rate within an instar is not just due to a constraint on oxygen supply, but that intrinsic demand decreases as well. COX activity decreases at the beginnings of successive instars, when constraints on oxygen supply are relieved, indicating a gradual decrease in the intrinsic demand for oxygen across instars.

### Midgut is a Constant Proportion of Total Biomass in Early Instars

If the relative mass of the midgut is a factor that contributes to the gradual decline in mass-specific metabolic rate, then the midgut should make up a progressively smaller proportion of total body mass as the larva increases in size. By contrast, if a decrease in mitochondrial activity accounts for the gradual decrease in the mass-specific metabolic rate, then we would expect specific activity of COX to decrease with body size.

In both the third and fourth larval instars, midgut mass represents 12–13% of total body mass (Fig. 3). Thus, in the early instars, the decrease in metabolic rate cannot be accounted for by a decrease in the proportion of this highly metabolically active tissue. In the fifth (final) larval instar, midgut mass decreases as a proportion of total body mass, in addition to a drop in COX activity, which suggests that in this instar a decrease in the proportion of highly metabolically active tissue likely contributes to the decrease in mass-specific metabolic rate. In the fifth instars, it is not clear which tissues occupy a relatively larger proportion of body mass, as the proportion of gut decreases. The fat body is a tissue that visibly increases as the larva progresses through the fifth instar, and could contribute to the observed pattern. The cuticle and tracheal walls thicken considerably (pers. obs.), thus increasing the non-metabolically active mass.

## Discussion

Across the third, fourth and fifth instars, the oxygen consumption rate of *Manduca* scales with mass 0.85 (Fig. 2), indicating a hypoallometric relationship. Previous studies showed that the CO_2_ emission rate of first, third and fifth instar *Manduca* caterpillars scaled with mass 0.98 [24], which is indistinguishable from isometric scaling. Sears et al. [13] likewise found that the overall metabolic exponent across all five instars of *Manduca* is about 0.95, but they also analyzed the scaling exponent for each instar individually and found these to be much smaller with a progressive decrease from instar to instar, and averaging about 0.66. Our results confirm that the mean metabolic scaling one obtains depends strongly on exactly when in ontogeny it is measured. Each instar has a different metabolic scaling, and even within an instar metabolic scaling declines after the larva passes the critical weight [23]. Thus an overall metabolic scaling exponent, taken across the entire ontogeny, obscures a complex and biologically interesting pattern of ontogenetic metabolic scaling. The exponent decreases from instar to instar (Fig. 1 and 2), and within an instar the exponent decreases after the middle of the instar when the larva passes the critical weight [23].

The hypoallometric ontogenetic scaling relationship of oxygen consumption in the fourth and fifth instars implies that the mass-specific metabolic rate decreases as the larva grows. Our results suggest that this decrease can be attributed to two factors. During the 5th larval instar there is a decrease in the relative mass of a highly metabolic tissue, the midgut (Fig. 3). In addition, during the 3rd, 4th and 5th larval instars the decline in mass-specific metabolic rate is associated with a decrease in specific COX activity (Fig. 4). Our findings on the decline in COX activity with respect to body mass are similar to those found in *Bombyx mori* [26], where the activity of COX per unit body mass decreased from instar to instar. The decreasing relative COX activity with increasing body mass helps to explain why the mass-specific metabolic rate decreases at the beginning of successive instars and suggests that decreasing intrinsic demand contributes to the hypoallometric scaling of metabolic rate with body mass.

It is clear from our findings that the scaling exponent for metabolic rate has different causes at different stages in development. Within an instar metabolic rate is initially demand-limited and scales with an exponent that declines from instar to instar. After the critical weight the metabolic rate is supply-limited and scales with an exponent of approximately 0 [23]. Thus if metabolic scaling is measured across an entire larval instar its value will depend, in part, on the relative durations of the pre-and post-critical-weight growth periods. The declining demand-driven metabolic rate from instar to instar is associated with a decline in the specific activity of COX. The exact pattern of this decline depends on how the relative activity of COX is measured: there is a rather smooth monotonic decreasing rate of decline when measured relative to body mass, and a non-monotonic decline when measured relative to protein content of the gut tissue (Figure 4). The latter case may be more informative and may indicate a progressive reduction in the density of mitochondria or a gradually increasing efficiency of ATP synthesis (for instance by a reduction in proton leakage) so that less oxygen is required for every unit of ATP produced. Whatever the mechanism will prove to be, it is clear that no single causal model can account for metabolic scaling during ontogeny.
